# Distributed hybrid-indexing of compressed pan-genomes for scalable and fast sequence alignment

**DOI:** 10.1371/journal.pone.0255260

**Published:** 2021-08-03

**Authors:** Altti Ilari Maarala, Ossi Arasalo, Daniel Valenzuela, Veli Mäkinen, Keijo Heljanko

**Affiliations:** 1 Department of Computer Science, University of Helsinki, Espoo, Finland; 2 Department of Computer Science, Aalto University, Espoo, Finland; 3 Helsinki Institute for Information Technology, Espoo, Finland; University of Nebraska-Lincoln, UNITED STATES

## Abstract

Computational pan-genomics utilizes information from multiple individual genomes in large-scale comparative analysis. Genetic variation between case-controls, ethnic groups, or species can be discovered thoroughly using pan-genomes of such subpopulations. Whole-genome sequencing (WGS) data volumes are growing rapidly, making genomic data compression and indexing methods very important. Despite current space-efficient repetitive sequence compression and indexing methods, the deployed compression methods are often sequential, computationally time-consuming, and do not provide efficient sequence alignment performance on vast collections of genomes such as pan-genomes. For performing rapid analytics with the ever-growing genomics data, data compression and indexing methods have to exploit distributed and parallel computing more efficiently. Instead of strict genome data compression methods, we will focus on the efficient construction of a compressed index for pan-genomes. Compressed hybrid-index enables fast sequence alignments to several genomes at once while shrinking the index size significantly compared to traditional indexes. We propose a scalable distributed compressed hybrid-indexing method for large genomic data sets enabling pan-genome-based sequence search and read alignment capabilities. We show the scalability of our tool, DHPGIndex, by executing experiments in a distributed Apache Spark-based computing cluster comprising 448 cores distributed over 26 nodes. The experiments have been performed both with human and bacterial genomes. DHPGIndex built a BLAST index for n = 250 human pan-genome with an 870:1 compression ratio (CR) in 342 minutes and a Bowtie2 index with 157:1 CR in 397 minutes. For n = 1,000 human pan-genome, the BLAST index was built in 1520 minutes with 532:1 CR and the Bowtie2 index in 1938 minutes with 76:1 CR. Bowtie2 aligned 14.6 GB of paired-end reads to the compressed (n = 1,000) index in 31.7 minutes on a single node. Compressing n = 13,375,031 (488 GB) GenBank database to BLAST index resulted in CR of 62:1 in 575 minutes. BLASTing 189,864 Crispr-Cas9 gRNA target sequences (23 MB in total) to the compressed index of human pan-genome (n = 1,000) finished in 45 minutes on a single node. 30 MB mixed bacterial sequences were (n = 599) were blasted to the compressed index of 488 GB GenBank database (n = 13,375,031) in 26 minutes on 25 nodes. 78 MB mixed sequences (n = 4,167) were blasted to the compressed index of 18 GB E. coli sequence database (n = 745,409) in 5.4 minutes on a single node.

## Introduction

Fast progress in High-throughput sequencing (HTS) technology has increased the sequencing throughput and decreased the whole-genome sequencing (WGS) price over a thousand-fold during the last 15 years [1]. At the same time, sequencing data volumes are growing several orders of magnitude, and the number of assembled whole-genomes increases rapidly as well. Storing, indexing, and searching genomic data requires a large amount of high-performance storage space, working memory, computing power, and network capacity. Read alignment and sequence matching are routine methods in many genomic studies. However, searching sequences from massive genomic databases is computationally intensive, and extremely so on whole-genome scale. To exploit the accumulating genomic data from genome research to clinical practice [2, 3] the efficient compression and indexing methods on large genomic data sets become an urgent need [4].

Computational Pan-genomics [5] exploits the information of multiple genomes in comparative analytics. Pan-genomic reference index constructed from multiple sequences can enable aligning of donor sequences to multiple genomes with a single shot instead of aligning sequences separately to every individual genomic index. Moreover, a pan-genomic reference can improve genetic variation discovery amongst populations by considering the genetic diversity of individuals and recombination [5–9]. Sherman and Salzberg [10] point out the importance and advantages of using pan-genomes in genetic variation studies instead of just a single reference genome.

Such a massive sequence data set as a pan-genome should be compressed to reduce storage space and to be accessed efficiently. Moreover, support for fast sequence matching and read alignment methods is critical when pan-genomes should be analyzed. Thus, compression methods should support random access to the compressed data. Developing text compression and indexing algorithms provide space and memory-efficient approaches to implement practical solutions for genomic data. Traditional Burrows-Wheeler Transformation (BWT) based self-indexes can provide such properties [11]. More sophisticated variants of self-indexes have been developed such as Lempel-Ziv factorization-based hybrid-indexes [12, 13], that better exploit the similarity between sequences to achieve significantly better compression ratios than traditional BWT indexes [11]. Hybrid-index combining Lempel-Ziv factorization allows sequences to be aligned to all the compressed sequences simultaneously together with tools such as BWA and Bowtie. Furthermore, the CHIC [14] aligner integrates the standard Bowtie and BWA read aligners with the hybrid-indexing method providing read alignment capability with compressed pan-genomes. In addition to the compression ratio improvements, the methods for creating the used indexes should also meet the scalability requirements with ever-growing sequencing data volumes and numbers of whole human genomes as well. Our focus is on constructing compressed hybrid-indexes that enable efficient sequence alignment to such indexes while still deploying conventional read and sequence aligners (Fig 4).

Traditional computational genome analysis algorithms and pipelines are developed for sequential data processing whereas high-performance computing has evolved towards parallel and distributed data processing for speeding up computation and analysis of massive data volumes. Moreover, current genome analysis tools and pipelines have been typically developed on demand by the researchers relying on existing sequential algorithms. This has led to that currently used bioinformatics pipelines and tools utilize a mixture of sequential algorithms making them often poorly scalable, computationally inefficient, inflexible, and inapplicable to distributed computing. Parallel and distributed computing frameworks, distributed filesystems and databases have been evolving while the price of storage and memory has been decreasing and now it is economically viable to move on to distributed computing.

In our previous studies, we have been focusing on the scalable assembling of reference pan-genomes for enabling pan-genomic variant calling utilizing hybrid-index [15] and scalable searching of viral sequences amongst numerous metagenomes assembled from human samples with ViraPipe [16]. Here, we focus on the distributed compressed hybrid-indexing and propose a scalable distributed compression and indexing tool for a massive number of assembled genomes with read alignment and sequence matching support. Our tool, DHPGIndex, is compatible with BWA [17] and Bowtie [18] legacy read aligners, and BLAST [19] sequence search tool. DHPGIndex is publicly available in GitHub (https://github.com/NGSeq/DHPGIndex).

## Materials and methods

The uncompressed size of the human reference genome is approximately 3 gigabases. Sequencing a single human genome with NGS can output Terabytes of read data, depending mainly on the sequencing coverage [20]. In order to assemble a reference genome or call variants based on a pan-genome, these short reads are aligned to every position in each individual in a reference pan-genome. The rapidly accumulating Whole-genome and NGS sequencing datasets make compression methods even more significant. However, to exploit compressed genomic data efficiently in analysis, the compression formats must support fast sequence searching and read alignment methods.

Human genetic variation between individuals is relatively low and, in addition, also individual genomes comprise a large number of repetitive sequences [21]. This property offers an excellent basis for compressing and representing collections of genomes very compactly. On the other hand, sequence searching requires that sequence is aligned base by base over the whole reference genome. For a human, the reference genome is over three billion base pairs long (resulting in over 3 GB of data per individual, e.g. GRCh37 assembly available from https://www.ncbi.nlm.nih.gov/assembly/GCF_000001405.13/).

Typical NGS analysis workflow is preceded by a read alignment process where billions of short read sequences from a donor sample can be aligned to a reference genome [22]. Sequence alignment compares short sequences to subsequences extracted from a genome. The similarity between the sequences is scored by giving a numerical cost for each different edit operation (substitution, insertion, or deletion). Then, we can measure the alignment quality and deduce the best matching sequence [23]. The alignment process generally needs indexing of a reference genome, to be able to efficiently compare a sequence to every position in each chromosome.

To enable sequence alignment with compressed pan-genomes, the individual genomes need to be indexed for an appropriate aligner. Typically, the amount of reference index data remains several magnitudes smaller than the read data sequenced from a donor genome, but with pan-genomes, the amount of index data grows in proportion to the number of individual sequences in the collection. A traditional alignment method would require indexing of each individual genome in the pan-genome and aligning of reads against every such index. This would result in that the amount of total main memory needed for the index during read alignment grows up to N x M where N is the number of sequences in the collection and M is the number of nodes in the cluster.

Burrows-Wheeler transformation (BWT) is an efficient method for constructing sequence indexes and enabling fast searching. Burrows-Wheeler transformation can be enhanced using a suffix-array for improving computational space- and time-efficiency [24]. Suffix-arrays are data structures representing long sequences with substrings where a substring occurs multiple times in the original sequence. Suffix-arrays can be constructed in linear time [24]. There are several Burrows-Wheeler index-based legacy tools for relatively fast read alignment such as BWA [17], Bowtie [18], and SOAP [25]. However, these tools do not utilize data compression techniques such as Lempel-Ziv [26] and are developed for sequential computing, and thus, not directly applicable to distributed systems. The index size can be reduced significantly with compressed hybrid-indexing methods by compressing the repetitive sequences between the genomes, that is, the human genome includes a large proportion of repetitive sequences which can be found from every individual. The compressed hybrid-index enables indexing of large pan-genomes as a whole that would require an unfeasible amount of memory with uncompressed sequences [13].

### Relative Lempel-Ziv compression

**Fig 1:**
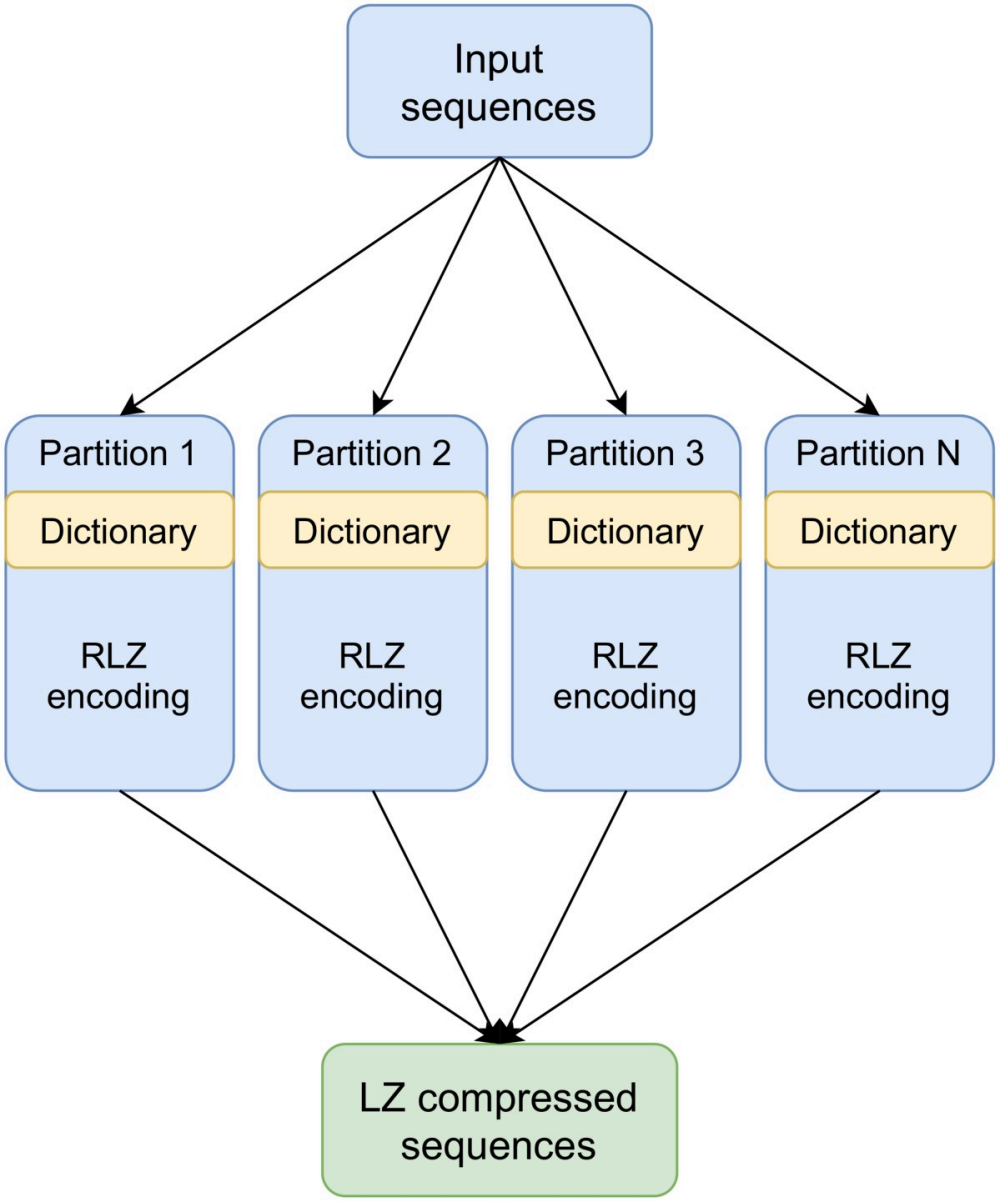
Distributed Relative Lempel-Ziv compression.

**Fig 2:**
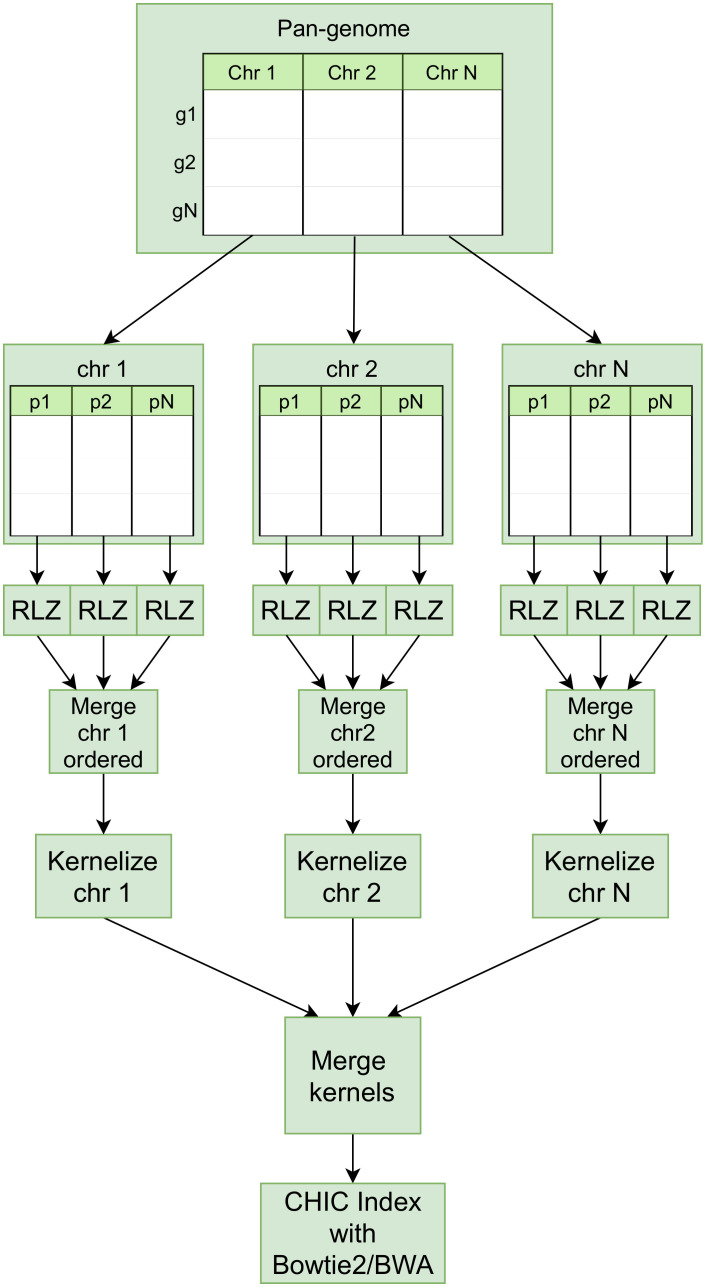
Distributed compression pipeline for hybrid-index.

Lossless data compression algorithms based on dictionary coding, such as Lempel-Ziv (LZ77) [26], enhanced by suffix-arrays are proven space- and time-efficient methods for compressing repetitive sequences [12, 27]. Relative Lempel-Ziv (RLZ) is a modification to the classical LZ77 [12] and is applicable to efficient distributed genome compression. Instead of applying an LZ77 sliding window method to limit the compression dictionary size, RLZ uses a prefix of the data as a dictionary. Valenzuela et al. [28] propose a CHICO indexer based on a hybrid-index implementation of LZ77 with kernelization [29] for compressing and indexing pan-genomes. Distributed RLZ method presented here extends the CHICO indexing by distributing and parallelizing the RLZ compression (Fig 1) and hybrid-indexing method (Fig 2).

Reference assembly methods typically require an index of the whole reference genome which has to be provided at the stage when raw reads are aligned to a reference. In the distributed setting, the reference index has to be provided as a whole for each alignment execution, meaning that during parallelized read alignment, the index has to be copied to every node in the computing cluster. Compressed self-indexing techniques using Lempel-Ziv and suffix trees have been already studied and found efficient for indexing repetitive sequences [12] which can facilitate space efficiency and reduce search space in the sequence alignment.

Valenzuela et al. propose the CHIC aligner [14] based on CHICO [28] and they have managed to index 200 human genomes with a compression ratio of 3:1 (540 GB of input data to 180 GB Bowtie2 index) in 35 hours with a very large memory single node having 1.5 TB RAM and 48 cores. There is also PanVC pipeline available from https://gitlab.com/dvalenzu/PanVC which uses the CHIC aligner to call variants from the pan-genomic index. Kuhnle et al. [30] propose an FM-index-based r-index tool for constructing a complete pan-genomic index. They were able to index 10 human genomes in 380 minutes with an index size of 36 GB on a single node with 10 cores (Intel(R) Xeon(R) CPU E5-2680 v2 @2.80 GHz) and 324 GB RAM. With 10 diploid genomes (58 GB) this results in an index compression ratio of 1.6:1.

In contrast, our distributed compression approach achieved an index compression ratio 532:1 with 1000 human haploid genomes (2890 GB). Our method utilizes distributed memory using less memory per node available and is thus more practical, e.g., for cloud computing.

### Kernelization

The kernelization method has been recently proposed by Gacie and Puglisi [29] for searching and indexing compressed genomic datasets. The kernel representation significantly reduces the repetition of identical sequences in the pan-genome where the repetitive sequences are parsed with LZ77 factorization using a dictionary that is constructed from the pan-genome prefix. That is, LZ factorization breaks down the repetitive sequences in the dictionary into suffix phrases (factors). Building the dictionary of the whole pan-genome would be too time-consuming and is not necessary due to repetitiveness, although, it can potentially improve the compression ratio. Kernelization uses suffix phrases factorized from the input sequence (here pan-genome) to represent the input sequence as a compact kernel sequence. To find all the occurrences of a query string in the original text, the positions of suffix phrase boundaries and phrase lengths in the original text are stored. The query sequences can be then searched from the kernel sequence and all the secondary matches to the original sequence can be found efficiently using advanced data structures [31].

### Hybrid-indexing

Hybrid-indexing was first presented by Ferrada et al. in [13] showing that the compressed self-index can be made compatible with conventional indexes. Hybrid-index is then accessible with conventional read aligners enabling fast sequence alignment to compressed index without losing aligner-specific properties. The repeating sequences in a pan-genome are compressed with RLZ relative to a sample dictionary parsed from the beginning of a pan-genome. The produced kernel sequence can be then indexed using conventional indexes such as Bowtie or BWA and used directly for read alignment with hybrid-index. CHIC [14] hybrid-index stores the original positions of suffix phrase boundaries and phrase lengths and uses auxiliary data structures to map the kernel alignments to the original pan-genomic positions. CHIC aligns reads against a single kernel sequence instead of aligning to each individual genome separately and the resulting kernel alignment is mapped back to the original genomic positions using the data structure of SDSL (https://github.com/simongog/sdsl-lite) library and its suffix-array binary searching capabilities over the suffix phrases. Read alignment is reported as a primary match which is the best scoring match of all possible matches and optionally as secondary matches that align to the other positions where an identical subsequence exists in the uncompressed pan-genome. The primary match can span over the multiple phrase boundaries while the secondary match can not and secondary matches are solved using the primary match as an input and searching the identical substrings over the LZ parse [32].

### Distributed computing frameworks for genomics

Apache Spark (http://spark.apache.org/) is an open-source framework developed for efficient iterative distributed large-scale computation in computing clusters. Computation in Spark is based on Resilient distributed datasets (RDD) [33], which are distributed and cached to working memory of multiple computing nodes in a cluster to be processed by tasks managed by YARN node managers. Each node then assigns an executor for local tasks which are run in parallel in multiple cores inside a node. Thus, data locality can be achieved in the nodes of the computing cluster and data processing done in parallel without reloading or moving any data.

The existing general genomics file formats are not designed for distributed file systems and especially binary formats BAM, BCF, and BED are not distributable without external tools. Hadoop-BAM [34] is a library originally developed for processing NGS data formats in parallel with both Apache Hadoop and Spark. Disq (https://github.com/disq-bio/disq) project is a continuation for Hadoop-BAM with better Spark compatibility. Hadoop-BAM can process distributed BAM and BCF files on Hadoop Distributed File System (HDFS) [35] in parallel and also in-memory with Spark. It includes the Hadoop Input/Output interface for distributing genomics file formats into HDFS and tools for, e.g., sorting, merging, and filtering of read alignments. Currently, supported genomics file formats are BAM, SAM, CRAM, FASTQ, FASTA, QSEQ, BCF, and VCF. Hadoop-BAM is already used in parallel genome analytics frameworks and libraries such as GATK4 (https://github.com/broadinstitute/gatk), Adam (https://github.com/bigdatagenomics/adam), Halvade [36], Seal [37] and SeqPig [38].

### Implementation

#### Distributed Relative Lempel-Ziv compression

We implement Distributed Relative Lempel-Ziv (DRLZ) compression (Fig 2) using Apache Spark for reducing the compression time through distributed parallelization. The RLZ compression method used in the DRLZ follows the RLZ method presented by Hoobin et al. [12] with the exception that each partition is encoded separately in parallel. The encoding part is implemented in Scala based on the kkp3 LZ factorization variant presented in [39, 40]. The dictionary part is extracted from the first N sequences in a partition and the dictionary is then used for compressing the rest of the sequences in a partition. In addition, a suffix-array of the dictionary is utilized for searching the matching suffixes from the dictionary more efficiently [12]. The suffix-array of the dictionary in each partition is constructed using a Scala implementation of algorithm presented by Kärkkäinen et al. [24]. The input sequence data is distributed to the HDFS and the distributed data is read into Spark DataFrame from the HDFS, decomposed into smaller partitions, and grouped by the partition identifiers. As human chromosomes are of different lengths, the data partitions of each chromosome can be kept close to the same size by changing the number of partitions. The limiting issue with Spark implementation is that the length of the dictionary is restricted by Java arrays roughly to 2 billion values (*Integer.MAX_VALUE*), however, the data decomposition enables us to use longer dictionaries within the partition and thus achieving a better compression ratio. Moreover, the data decomposition allows us to control peak memory usage and the workload balance by keeping the partition size constant. To improve the compression ratio with mixed species (e.g., bacteria or viruses), the sequences are grouped by their taxonomies and the most complete assemblies (e.g., whole genomes and chromosomes) are selected primarily for the dictionary. The encoded partitions are written in binary format into HDFS and the order of the partitions is maintained by using numbered file names. The user should be aware that searching for secondary matches is limited inside the partition as the LZ phrases are not linked across the partitions. If all the secondary matches should be found, one has to configure the aligner to search for approximate matches, e.g., with bowtie2 -a option.

#### Distributed hybrid-indexing

**Fig 3:**
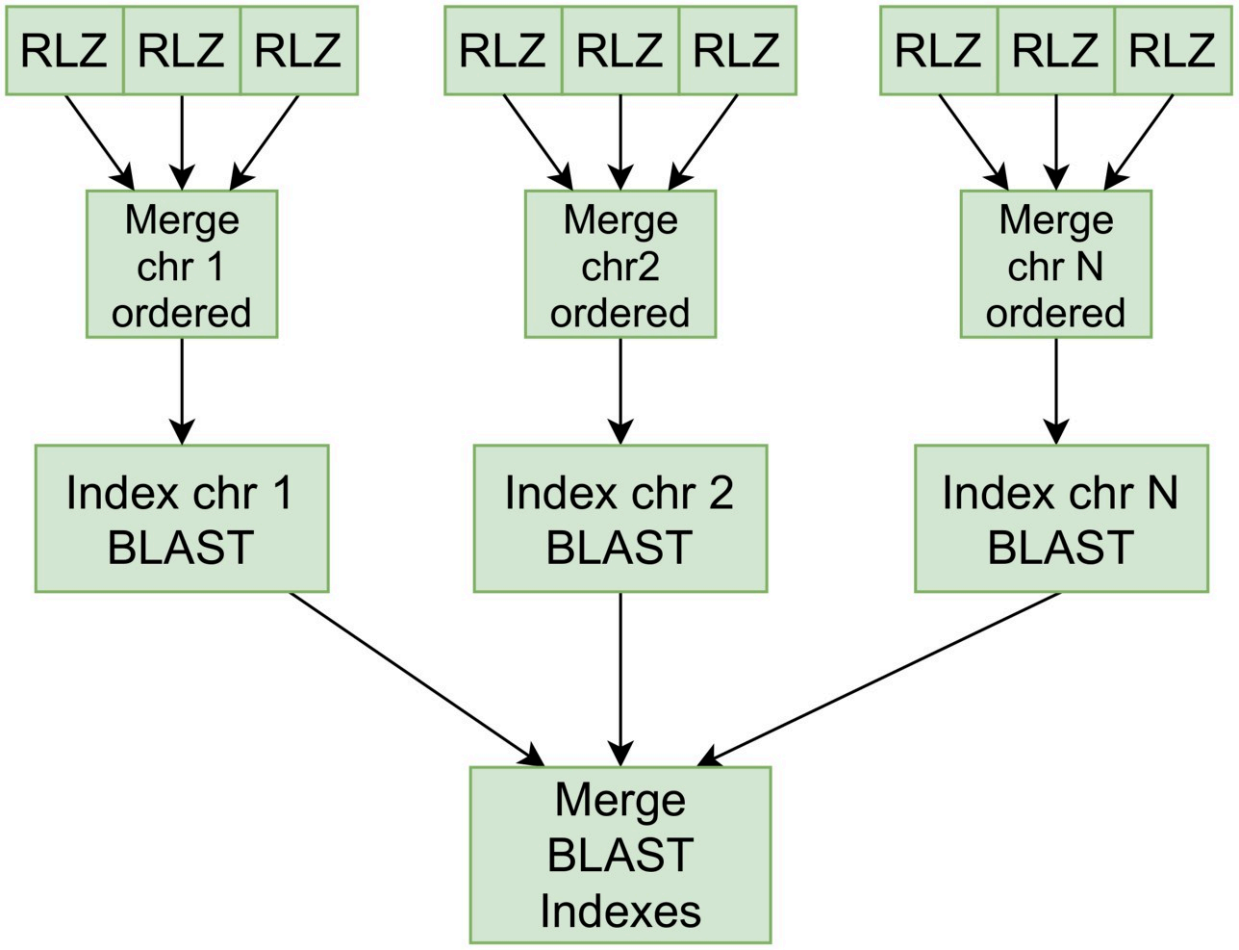
Distributed hybrid-indexing with BLAST.

The RLZ compressed data partitions are eventually merged and downloaded from the HDFS to distributed nodes where the kernel representations are composed with CHIC in parallel per partition. To construct the complete Bowtie index with human genomes, the chromosomal partitions are kernelized in parallel and the kernel sequences are merged into a single file and indexed as a whole. For the distributed indexing option, we have implemented tools for CHIC to merge the metadata files that it uses in the alignment phase to map kernel-aligned sequences back to the original genomic positions. The BLAST indexer input size is limited to 4 GB, thus with large pan-genomes we construct separate BLAST indexes from partial kernels and link the indexes into a single searchable index with blastdb_aliastool (Fig 3).

CHIC supports legacy BWA and Bowtie2 indexes for FASTQ read alignment with the compressed index, and FM indexes for full-text searching [14]. The Bowtie2 read aligner is recommended to be used with pan-genomes as it can index arbitrary length sequences. In addition, we have extended CHIC to compress BLAST indexes for searching FASTA formatted sequences with the BLAST tool. CHIC indexer takes a maximum query length (there is no upper bound for this parameter) as an input parameter that should be chosen carefully as it affects the compression time and the compression ratio, and can affect the alignment accuracy. BLAST searches initially small fractions of the query sequence defined by the word_size parameter which is 28 bases with megablast and 11 bases with blastn in default, thus it is likely to find similar sequences even though maximum query length is smaller than the length of the query sequence. That is, if the alignment is longer than the maximum query length the secondary match may be omitted. With the read aligners, one should choose the maximum query length based on the maximum read length. The performance-wise rule is: the longer the maximum query length the worse the compression ratio and the shorter the compression time. However, the compression ratio depends on the compressed sequence length and its content at the first hand.

#### Sequence alignment with RLZ compressed hybrid-index

**Fig 4:**
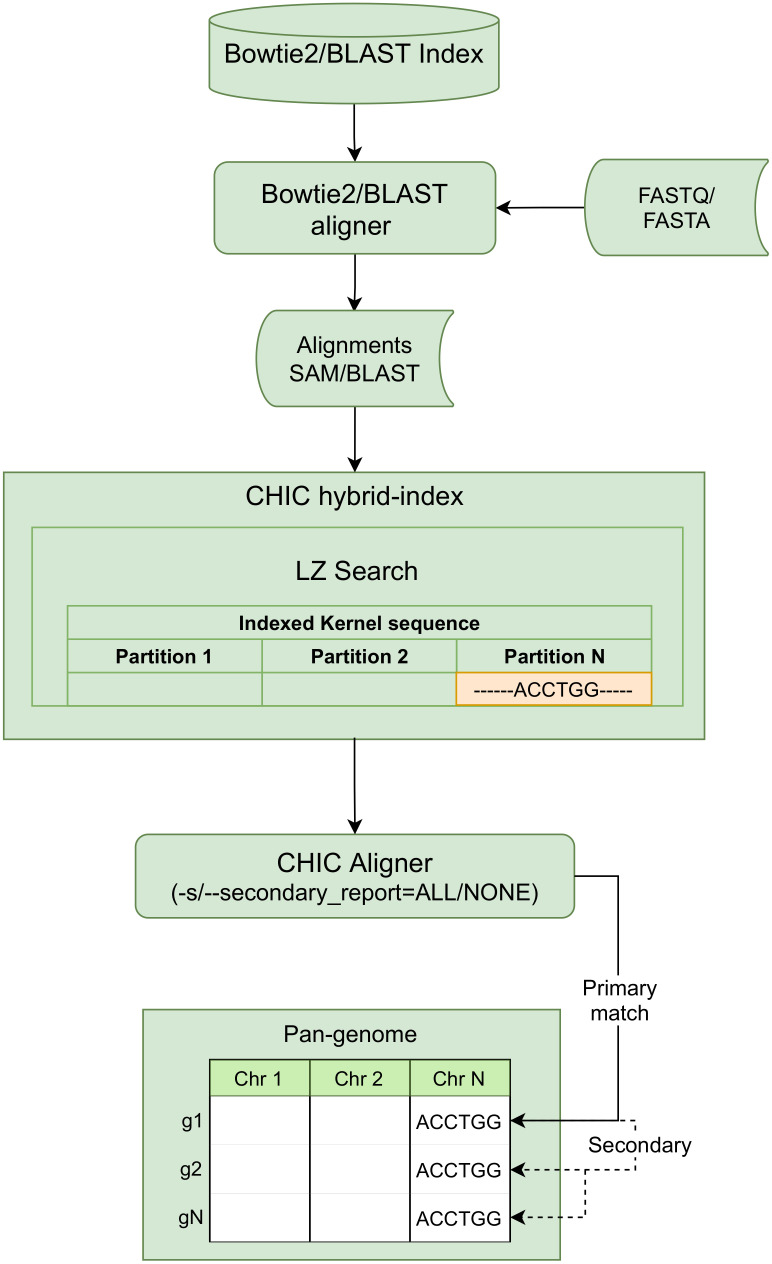
Sequence alignment with hybrid-index.

CHIC aligns sequences against a single indexed kernel sequence generated from the pan-genome instead of aligning to each individual genome separately (Fig 4). The CHIC aligner integrates BWA, Bowtie2, and BLAST aligners and uses them to align FASTQ or FASTA sequences to the kernel sequence indexed by an appropriate indexer. Eventually, the kernel-aligned sequences are mapped back to the original genomic positions using the CHIC index’s internal data structures with LZ compressed sequences. The CHIC aligner can be executed with different parameter settings to find primary matches only (default), to find primary and secondary matches (-sALL), and with bowtie2 -all option (reports all approximate alignments) to find primary+secondary matches also from approximate read alignments (Fig 4). The integrated Bowtie2 aligner can be configured using the original Bowtie2 parameters, and it may be useful to restrict the number of approximate alignments, e.g., with bowtie2 -k parameter in real use cases. The integrated BLAST search can be configured with the original BLAST search parameters and one may find it useful to search matches by setting threshold values such as evalue, pident, and max_hsps. A user should be aware with the distributed indexing that the secondary matches can be reported only from the same partition that the primary match is aligned to, i.e., not from the other chromosome. However, the omitted secondary matches can be covered by searching the approximate alignments.

### Experiments

#### Data preparation

The collection of genomes (i.e., the pan-genome) itself is composed of assembled genomes. We experiment both with human genomes and with bacterial genomes. We generate a human pan-genome of whole-genomes based on GRCh37 (NCBI accession number GCF_000001405.13) human reference genome by applying SNPs from phased haploid VCF data to GRCh37 reference thus generating two consensus haploids per individual into a pan-genome. Pan-genome is generated from 1000 Genomes phase3 autosomal (chromosomes 1-22) VCF data including 2506 individuals in total available from ftp://ftp.1000genomes.ebi.ac.uk/vol1/ftp/release/20130502/. Each genome in a collection is assembled by applying subsets of variants from VCF files to a standard reference genome with vcf2multialign (https://github.com/tsnorri/vcf2multialign) tool and loading produced FASTA files into HDFS under the same folder. The FASTA files are separated by sample id and chromosome numbers for enabling the distributed indexing and flexible data management. When diploid genomes (e.g., humans) are assembled with vcf2multialign, it generates two sequences, both haploids per genome. Each subset used in our experiments is a superset of a larger set, e.g., subset n = 250 is contained in the n = 500 dataset.

With the Bowtie2 read aligner, we use next-generation sequencing (NGS) reads. Whole-exome sequencing data sequenced from single individuals (1000 genomes sample NCBI SRA accession numbers SRR765989 and SRR233147) is used in the human pan-genome read alignment experiments. In the BLAST sequence search experiment with the human pan-genome, we search CrispR-Cas9 associated guide RNA target sequences [41] downloaded from http://arep.med.harvard.edu/human_crispr including 189,864 sequences of length 23 base pair each.

Bacterial sequences were downloaded from the NCBI assembly database (https://www.ncbi.nlm.nih.gov/assembly/). The complete bacterial genomes and scaffolds were included generating 488 GB of FASTA data in total in total comprising of 13,375,031 sequences available from https://doi.org/10.5281/zenodo.4958582, https://doi.org/10.5281/zenodo.4958584, https://doi.org/10.5281/zenodo.4955279, and https://doi.org/10.5281/zenodo.4958582. Escherichia coli RefSeq sequences were downloaded including 745,409 sequences (all assembly types) generating 18 GB dataset (available from https://doi.org/10.5281/zenodo.4926006). The E.coli pan-genome was constructed by simply loading all the individual sequences in FASTA format into HDFS under the same folder. The 488 GB bacterial data set is split into 25 partitions and loaded into separate HDFS folders so that the distributed indexing can be managed flexibly on all 25 computing nodes.

The next-generation sequencing reads used in bacterial experiments are sequenced from Escherichia coli isolates (NCBI SRA accession number SRR13657535) and from a food-based metagenomic WGS sample spiked with the synthetic microbial community (NCBI SRA accession number ERR3079359). Query sequences used in the BLAST experiment with 488 GB bacterial pan-genome are randomly selected subsets of a pan-genome the sequences are aligned to. The query subset available from https://doi.org/10.5281/zenodo.4980557 includes 599 sequences (30 MB) with mean sequence length of 50342 bp, minimum length of 235 bp, and maximum length of 1469740 bp. The query sequences used with E.coli pan-genome are manually selected assemblies from NCBI RefSeq sequences including E.coli, Streptococcus and Klebsiella strains. The query subset used with the E.coli pan-genome available from https://doi.org/10.5281/zenodo.4980557 includes 4,167 sequences (78 MB) with mean sequence length of 19399 bp, minimum length of 179 bp, and maximum length of 989273 bp.

#### Computing environment

We utilize the computing resources of the Finnish IT Center for Science (CSC) in our experiments. We run the experiments on the Apache Spark cluster in a cloud computing environment. The cluster consists of 25 Spark worker nodes having 40 GB of RAM and 16 virtual cores (Intel(R) Xeon(R) CPU E5-2680 v3) in each and one Spark master node having 256 GB of RAM and 48 cores. The whole cluster comprises 448 CPU cores, 1.256 TB of RAM, InfiniBand 40 GB/s network, 30 TB of HDD storage space in total.

## Results

### Compression and indexing

First, we test the effect of reference dictionary length to the compression ratio with n = 250 human haploid genomes (772.5 GB) by increasing the RLZ dictionary length from 1% to 45% of the total pan-genome (Table 1). The results show that the compression ratio increases in proportion to the dictionary size. The compression time increases 3.4 times while the compression ratio increases 27.6 times from 1% to 45% dictionary lengths. In the following experiments, we choose to use 30% dictionary length as a compromise to keep the compression time tolerable with the longer pan-genomes. With human pan-genomes, the chromosomes are split to closely similar size data partitions by decreasing the number of partitions from 30 to 8 while increasing the chromosome number from 1 to 22.

**Table 1:** Compressing a human pan-genome (n = 250) with increasing RLZ dictionary length (% of pan-genome length).

Dict.length (%)	DRLZ (min)	Compressed size (GB)	CR	Bpc
1	111	52.2	13.8	0.072
15	269	4.8	150.5	0.0066
30	326	2.8	258.0	0.0039
45	398	1.9	380.3	0.0026

Secondly, we test the scalability of Distributed RLZ compression with different pan-genome sizes ranging from n = 250 to n = 1000 haploid genomes. The compressed size equals the kernel size which is indexed in the following experiments. Table 2 shows the compression result with accumulating pan-genome size. The total runtime with n = 250 is 326 minutes, 678 minutes with n = 500, 1049 minutes with n = 750, and 1421 minutes with n = 1000. Compression ratios are 258:1, 208:1, 187.4:1 and 176.8:1 respectively. Bytes per character (Bpc) is simply the inverse of the compression ratio.

**Table 2:** Compressing human pan-genomes using reference sequence size of 30%.

n	Size (GB)	DRLZ (min)	Compressed size (GB)	CR	Bpc
250	772.5	326	2.8	258.0	0.0039
500	1445	678	6.8	208.0	0.0048
750	2217.5	1049	11.6	187.4	0.0053
1000	2890	1421	16	176.8	0.0057

**Fig 5:**
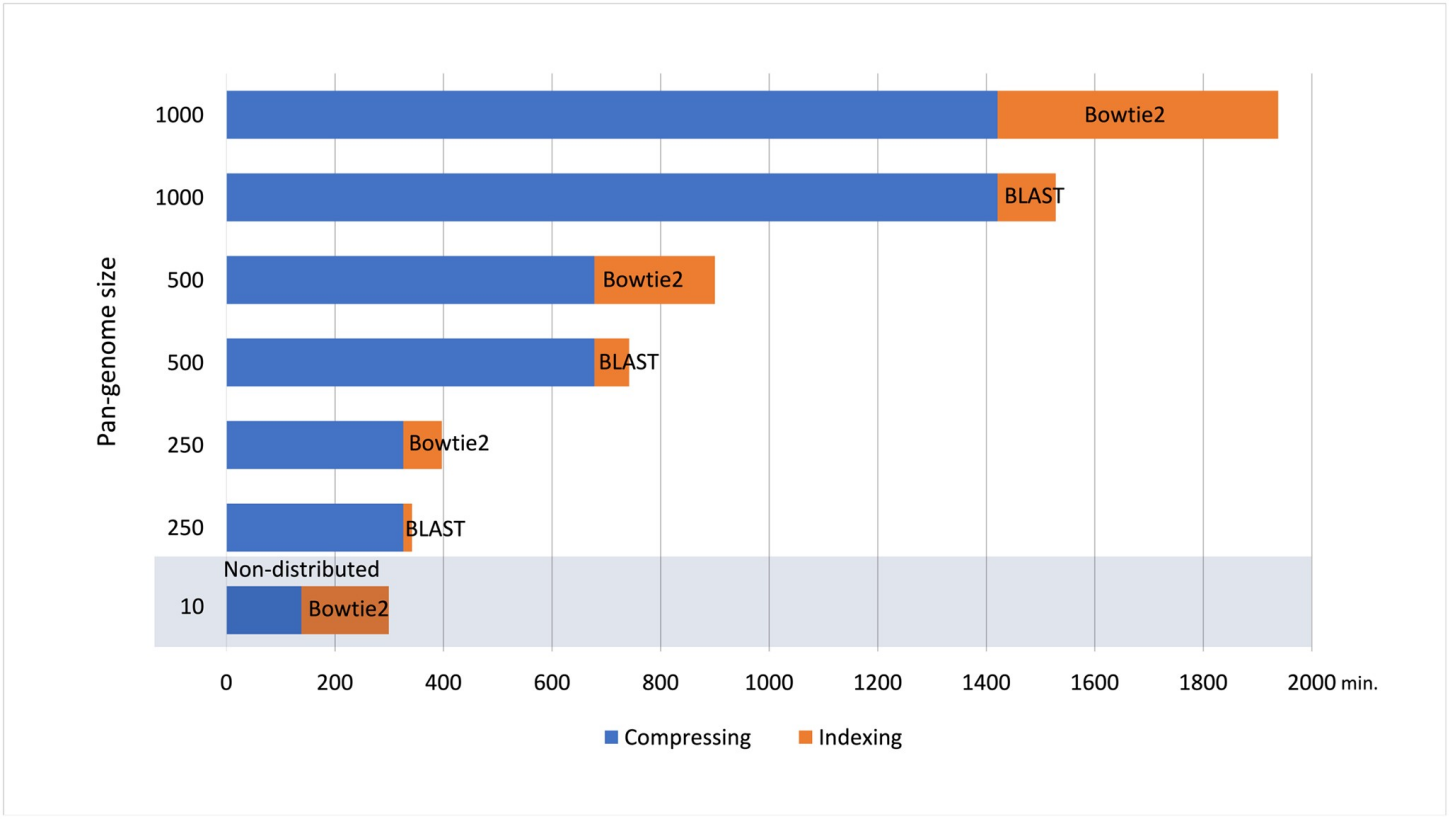
Summary of compressing and indexing complete human pan-genomes with distributed and non-distributed methods.

Third, we construct complete indexes for compressed pan-genomes of sizes n = 500 and n = 1000 compatible with Bowtie2 and BLAST (Table 3). Here, the indexing time includes also the distributed kernelization step using RLZ compression from the DRLZ step. The index size includes the index generated by the standard tool and the data generated by the CHIC. Bowtie2 index is constructed from a single kernel sequence concatenated from the chromosomal kernels on a single node. The BLAST index is constructed from chromosomal kernels on 22 nodes and the BLAST database is concatenated from the chromosomal indexes with blastdb_aliastool due to its limited input size (4 GB). Fig 5 summarizes the DRLZ compression and distributed indexing execution times with different pan-genome sizes. Then n = 250 subset was indexed in 71 minutes with Bowtie2 and in 16 minutes with BLAST with compression ratios of 157:1 and 870:1 respectively. The n = 500 subset was indexed in 222 minutes with Bowtie2 and in 64 minutes with BLAST with compression ratios of 87:1 and 542:1 respectively. The n = 1000 subset was indexed in 517 minutes with Bowtie2 and in 107 minutes with BLAST with compression ratios of 76:1 and 532:1 respectively. To note, the distributed kernel sequence construction time observed was 62 minutes for n = 500 and 104 minutes for n = 1000 with both tools, and the rest of the indexing time was spent in the index construction with the used tool. The Bowtie2 input kernel sequence size was 2.8 GB for n = 250, 6.8 GB for n = 500 and 16 GB for n = 1000. The BWA was not able to index the same data sets. The CHIC indexer has been run with the maximum query length parameter of 102 base pairs which covers the maximum sequence length of the test datasets used in the alignment experiments. As a reference, we compress and index a human pan-genome (n = 10) with the original CHIC tool (non-distributed in Fig 5). Non-distributed RLZ compression took 138 minutes and Bowtie2 indexing 161 minutes on a single node using 16 cores resulting in index compression ratio of 1.67:1.

**Table 3:** Building a complete hybrid-index for a human pan-genome using distributed indexing.

n	Size (GB)	Tool	Indexing (min)	Index (GB)	CR	Bpc
250	722.5	Bowtie2	71	4.6	157.07	0.00637
250	722.5	BLAST	16	0.83	870.48	0.00115
500	1445	Bowtie2	222	16.2	87.03	0.0115
500	1445	BLAST	64	2.6	542.31	0.00184
1000	2890	Bowtie2	517	38	76.05	0.0131
1000	2890	BLAST	107	5.3	532.08	0.00188

Bowtie2 index is created on a single node. BLAST index is created in parallel on 22 nodes from chromosomal kernels. The kernelization step is included in the indexing time and it has been performed in parallel on 22 nodes in both cases.

Fourth, we test distributed RLZ compression and indexing of mixed bacterial populations (Table 4). Compressing 18 GB E.coli data set took 33 minutes using the whole computing cluster. Indexing the compressed E.coli data set on a single node took 2 minutes resulting in a compression ratio of 101.69:1 with BLAST and 8 minutes with Bowtie2 resulting in a compression ratio of 50.56:1. With the GenBank 488 GB data set we use distributed indexing method and harness all 25 computing nodes for indexing. 488 GB GenBank data set was DRLZ compressed and indexed with BLAST in 575 minutes resulting in CR 61.78:1 and in 609 minutes with Bowtie2 resulting in CR 10.61:1. The DRLZ compression time was 551 minutes with the 488 GB mixed data set, where the distributed kernelization took 23 minutes, and the distributed BLAST indexing took only 1 minute. With the Bowtie2, the CHIC indexer has been executed with the maximum query length parameter of 502 base pairs which covers the maximum read length of the test datasets used in the alignment experiments. In the BLAST experiments, the query length parameter of 1000 bp (the parameter sets the maximum supported length for the query sequence) has been used which was assumed to cover most of the aligned sequence lengths.

**Table 4:** DRLZ compression and indexing of bacterial pan-genomes with BLAST.

Tool	Pan-genome (size)	Seqs.	DRLZ	Indexing	Index size	CR	Bpc
BLAST	*E*.*coli* (18 GB)	745k	33 min	2 min	0.177 GB	101.69	0.0098
BLAST	GenBank (488 GB)	13.4 M	551 min	24 min	7.9 GB	61.78	0.0162
Bowtie2	*E*.*coli* (18 GB)	745k	33 min	8 min	0.356 GB	50.56	0.0198
Bowtie2	GenBank (488 GB)	13.4M	551 min	58 min	46 GB	10.61	0.0943

Bowtie2 index is created on a single node from concatenated kernel. BLAST index is created in parallel on 25 nodes from distributed kernels. The kernelization step is included in the indexing time.

### Searching and read alignment

First, we align sequences to a compressed index of human pan-genome (n = 1000) with different tools, see Table 5. Read alignment is performed on a single node (16 cores) with the paired-end read data sequenced from a single individual. The CHIC aligner finds only primary matches as a default which is used in the experiments unless otherwise stated. Bowtie2 aligned 14.6 GB of paired-end reads to compressed index of size n = 1000 in 31.7 minutes. BLAST search is tested by aligning 189,864 Crispr-Cas9 gRNA target sequences of length 23 base pairs each [41] (23 MB Fasta data in total) to the compressed BLAST index of 1000 human haploid genomes. The BLAST search is performed in parallel per chromosomal partitions on 22 distributed nodes (16 cores in each) with parameters: -task megablast -word_size 23. Megablast is used as we want to find exact matches of Crispr-Cas9 target sequences and it is intended to find highly similar sequences. Crispr-Cas9 target sequence BLAST search took 45 minutes. As a reference, the same sequence alignment to a single uncompressed GRCh37 reference genome index took 15 minutes with Bowtie2, and 7 minutes with BLAST using a single node and 16 cores. The secondary matches are not relevant to be reported in most use cases. However, the effect of searching all the matches with different CHIC aligner parameters from a human pan-genome is demonstrated in Table 6. The number of mapped reads grows greatly when secondary matches with the -sALL option are included, and especially so when the Bowtie2 is set to search also approximate alignments (with bowtie2 -a option).

**Table 5:** Aligning sequences to compressed human pan-genome index of size n genomes.

Tool	n	Query sequences	min	Mapped
Bowtie2	1000	2x28.86M (2x7.3 GB)	31.7	10.12M
BLAST(megablast)	1000	189.9k (23 MB)	45.2	639k

**Table 6:** The effect of CHIC aligner parameters to the number of pan-genome mapped reads (n = 10).

		Mapped (min)		
n	Reads (size)	default	sAll	bowtie2 -a
10	2x14.69M (2x949 MB)	0.126M (0.6)	530.14M (12.3)	8154.46M (302)

The CHIC aligner with Bowtie2 was run with three different parameter settings: default to find primary matches only, -sALL to find primary+secondary matches, and bowtie2 -a option (reports all approximate alignments) to find primary+secondary matches also from approximate read alignments.

Secondly, we test alignment performance with the index generated from bacterial populations (Tables 7 and 8). With the E.coli 18 GB pan-genome, the alignment experiment is performed on a single node (16 cores). With the GenBank 488 GB data set, the alignment experiment is performed in parallel per database partition on 25 distributed nodes (16 cores in each). Searching primary matches for 4,167 (78 MB) sequences from the 18 GB compressed E.coli index took 5.38 minutes outputting 1741k matches. Searching all the matches with chic_align -sALL parameter (includes primary and secondary matches) for the same sequences took 7.55 minutes outputting 1872k matches. Searching primary matches for 599 (30 MB) randomly selected GenBank sequences from the 488 GB compressed GenBank index took 26.17 minutes while searching all the matches with chic_align -sALL option (includes primary and secondary matches) for the same sequences took 44.56 minutes. 43.60 million matches were found in total from which 13.79 million matches were primary matches. BLAST task blastn was run with 16 threads using default parameters. As the alignment quality is not restricted by any constant value as default, the same sequence can align to several positions and several reference sequences resulting in a large number of matches. As a reference, the same BLAST search of 4.2k sequences from a collection of 27.8k (1 GB) E.coli sequences using a conventional BLAST index took 8.9 minutes on a single node with 16 cores.

**Table 7:** Aligning bacterial sequences to a compressed index with BLAST (blastn).

		Matches(min)	
Pan-genome (seqs., size)	Query seqs. (size)	primary	-sALL
*E*.*coli* (745k, 18 GB)	4.2k (78 MB)	1741k (5.38)	1872k (7.55)
GenBank (13.4M, 488 GB)	599 (30 MB)	13.79M (26.17)	43.60M (44.56)

The CHIC aligner was run with two different parameter settings: default to find primary matches only, and -sALL to find primary+secondary matches.

**Table 8:** Aligning next-generation sequencing reads to a compressed index with Bowtie2.

		Mapped (min)		
Pan-genome (seqs., size)	Reads (size)	default	-sAll	bowtie2 -a
*E*.*coli* (745k, 18 GB)	3.1M (792 MB)	73 (5.94)	73 (5.98)	2.2k (31.47)
GenBank (13.4M, 488 GB)	27.2M (4334 MB)	1.07k (12)	41.5k (24)	228.4k (92)

The CHIC aligner has been executed with three different parameter settings: default to find primary matches only, -sALL to find primary+secondary matches, and bowtie2 -a option (reports all approximate alignments) to find primary+secondary matches also from approximate read alignments.

With the Bowtie2 read aligner, we use next-generation sequencing reads. 3.1 million reads were aligned (NCBI SRA accession number SRR13657535) to E.coli pan-genome in 5.94 minutes with default parameters resulting in 73 mapped reads, with –secondary_report = ALL (-sALL) option in 5.98 minutes resulting in 73 mapped reads, and in 31.47 minutes by executing bowtie2 -a option resulting in 548 mapped reads. 27.2 million reads (NCBI SRA accession number ERR3079359) were aligned to GenBank pan-genome in 12 minutes with default parameters resulting in 1.07k mapped reads, in 24 minutes sALL parameter resulting in 41.5k mapped reads, and in 92 minutes by executing bowtie2 with -a parameter resulting in 228.4k mapped reads.

## Discussion

In this work, we design and implement a distributed version of the compressed hybrid indexing method based on Relative Lempel-Ziv (RLZ) compression for scaling pan-genomic read alignment and sequence search to perform on large pan-genomes in practice. We have experimented with the Distributed Relative Lempel-Ziv (DRLZ) compression and distributed hybrid indexing methods in Apache Spark computing cluster comprising 448 cores distributed on 26 nodes. The developed DHPGIndex tool has been tested with human and bacterial genomes but can be used with any species with little effort. The experiments have been conducted using different settings and input data sets for demonstrating the performance and scalability of our methods.

With the increasing number of genomes, the distributed DRLZ compression scales quite well with the 30% dictionary length (Table 2). The effect of dictionary size is experimented with 250 genomes (Table 1) by increasing the dictionary size from 1% to 45%. The DRLZ compression ratio increases in proportion to the dictionary length and the compression time increases only 3.4 times while the compression ratio increases 27.6 times from 1% to 45% dictionary length. This is because that a longer reference dictionary sequence contains more repetitive subsequences with the rest of the pan-genome, and thus, increases the compression ratio. The 30% dictionary size was chosen as a compromise between the compression time and the compression ratio in the experiments.

BLAST indexing step with human pan-genome is distributed by chromosomes and the chromosomal indexes are merged. We were able to compress 1000 genomes index with a compression ratio of 532:1. Distributed BLAST indexing time is negligible compared to kernelization time which was 104 minutes with 1000 genomes. With Bowtie2 the pan-genome was kernelized in chromosomal partitions in parallel and the generated kernels were merged for indexing on a single node as a whole. By using longer dictionary sequences, the compressed distributed kernel sequence size stays below the maximum input sequence length limited by the BLAST (4 GB) indexer. Moreover, the longer dictionary increases the compression time but decreases the indexing time due to the shorter kernel sequence.

The limiting issue in our current implementation to use whole human genomes as a dictionary with Spark, is that the length of the dictionary is restricted by Java array which can hold up to roughly 2 billion (*Integer.MAX_VALUE*) values. Additionally, a long dictionary uses more memory while compressing. Thus, to improve the compression ratio while keeping the peak memory usage low, we split each chromosome into N partitions and increase the number of dictionary reference sequences in each partition.

The compression ratio of the mixed bacterial population is lower than with the human genomes. That is, the DNA sequences between bacterial genomes vary greatly and less repetition is present. By grouping the bacterial sequence by their taxonomies, the compression ratio improved slightly but stays still relatively low compared to the compressed human genome. In the near future, we will study alternative methods, such as sequence clustering to improve compression ratio with microbial genomes.

The alignment performance depends greatly on the number of the matched sequences thus we observe a great increase in alignment time when secondary matches are included as human pan-genome comprises highly similar sequences (Table 6). With dissimilar bacterial sequences the alignment time increases only slightly due to few or zero additional matches (Tables 7 and 8).

Altogether, our distributed DHPGIndex (https://github.com/NGSeq/DHPGIndex) tool enables now compressing and indexing large pan-genomes in a tolerable time in practice. The compressed pan-genomic hybrid-index can be directly used with read aligners such as Bowtie2 and BWA or with the BLAST search tool.
